# Comparative Mitogenomes of Two *Coreamachilis* Species (Microcoryphia: Machilidae) along with Phylogenetic Analyses of Microcoryphia

**DOI:** 10.3390/insects12090795

**Published:** 2021-09-05

**Authors:** Jia-Yin Guan, Shi-Qi Shen, Zi-Yi Zhang, Xiao-Dong Xu, Kenneth B. Storey, Dan-Na Yu, Jia-Yong Zhang

**Affiliations:** 1 College of Chemistry and Life Science, Zhejiang Normal University, Jinhua 321004, China; a1345413239@163.com (J.-Y.G.); shenshiqi0824@163.com (S.-Q.S.); cixi55@126.com (Z.-Y.Z.); KI01110015@163.com (X.-D.X.); ydn@zjnu.cn (D.-N.Y.); 2 Department of Biology, Carleton University, Ottawa, ON K1S 5B6, Canada; kenneth.storey@carleton.ca; 3 Key Lab of Wildlife Biotechnology, Conservation and Utilization of Zhejiang Province, Zhejiang Normal University, Jinhua 321004, China

**Keywords:** *Coreamachilis*, Microcoryphia, mitochondrial genome, phylogenetic relationship, selection pressure

## Abstract

**Simple Summary:**

Bristletails (Insecta: Microcoryphia) are primarily wingless insects, some of which have been found to exhibit parthenogenesis. In the genus *Coreamachilis*, parthenogenesis occurs in *C. coreanus*, whereas sexual reproduction is found in *C. songi*. Therefore, after obtaining mitochondrial genome sequences of these two species, we analyzed their selection pressure, based on phylogenetic trees of Microcoryphia. However, no positive selection was found in the mitochondrial protein coding genes of either *C. coreanus* or *C. songi*. In addition, a long hairpin structure was found between ND1 and 16S rRNA genes in Machilinae and Petrobiinae, which was highly consistent with the phylogenetic results.

**Abstract:**

The order Microcoryphia, commonly known as bristletails, is considered as the most primitive one among living insects. Within this order, two species, *Coreamachilis coreanus* and *C. songi* (Machilidae: Machilinae), display the following contrasting reproductive strategies: parthenogenesis occurs in *C. coreanus*, whereas sexual reproduction is found in *C. songi*. In the present study, the complete mitogenomes of *C. coreanus* and *C. songi* were sequenced to compare their mitogenome structure, analyze relationships within the Microcoryphia, and assess adaptive evolution. The length of the mitogenomes of *C. coreanus* and *C. songi* were 15,578 bp and 15,570 bp, respectively, and the gene orders were those of typical insects. A long hairpin structure was found between the ND1 and 16S rRNA genes of both species that seem to be characteristic of Machilinae and Petrobiinae species. Phylogenetic assessment of *Coreamachilis* was conducted using BI and ML analyses with concatenated nucleotide sequences of the 13 protein-coding genes. The results showed that the monophyly of Machilidae, Machilinae, and Petrobiinae was not supported. The genus *Coreamachilis* (*C. coreanus* and *C. songi*) was a sister clade to *Allopsontus helanensis*, and then the clade of ((*C. coreanus* + *C. songi*) + *A. helanensis*) was a sister clade to *A. baii*, which suggests that the monophyly of *Allopsontus* was not supported. Positive selection analysis of the 13 protein-coding genes failed to reveal any positive selection in *C. coreanus* or *C. songi*. The long hairpin structures found in Machilinae and Petrobiinae were highly consistent with the phylogenetic results and could potentially be used as an additional molecular characteristic to further discuss relationships within the Microcoryphia.

## 1. Introduction

Bristletails (Insecta: Microcoryphia) are primarily wingless insects, living in places such as under fallen leaves, the bark of trees, moss on wet logs, etc. They can be found at the seashore or inland all over the world. There are over 500 species in two families (Machilidae and Meinertellidae), and three subfamilies of Machilidae (Petrobiellinae, Petrobiinae, and Machilinae) have been reported [1,2]. The genus *Coreamachilis* belongs to the subfamily Machilinae. At present, there are two reported species of *Coreamachilis*; *C. coreanus* is distributed in Northern Korea and China [3], along with *C. songi* in China [4]. When Mendes described *C. coreanus* from North Korea, he did not find any male individuals, suggesting that parthenogenesis occurred in *C. coreanus* [5]. By contrast, *C. songi* was reported to show sexual reproduction [4].

Many studies have reported parthenogenetic lineages, particularly among species living at higher altitudes or in extreme habitats [6], indicating an association between parthenogenesis and environmental selection pressures that lead to specialized life cycles [7]. It was suggested that the loss of sexual reproduction was driven by a very general selective force [8], and selective factors promoted by variations in environmental conditions may be advantageous to parthenogenic reproduction [9]. By contrast, sexual reproduction is common among animals, and there have been many studies suggesting that natural selection can affect the characteristics of sexual reproduction [10,11]. In addition, parthenogenesis is rare in Microcoryphia [5]. Hence, we wondered if the mechanism of parthenogenesis in *C. coreanus* is under selective pressure. Also, we aimed to determine if there is a relationship between sexual reproduction and parthenogenesis within *Coremachilis,* related to selective pressure.

Insect mitogenomes are usually double-stranded circular molecules with a length of 14–20 kb, encoding 13 protein-coding genes (PCGs), 22 transfer RNAs (tRNAs), two ribosomal RNAs (rRNAs), and a control region (CR; or the AT-rich region) [12,13]. Because of their characteristics of fast evolution rates, small genome sizes, and low sequence recombination, mitogenomes have been used widely as molecular markers for phylogenetic analyses [13,14,15]. Many studies have now shown that specific gene rearrangements can occur in the mitogenomes of different families or genera [16,17,18]. Intergenic regions can also be used as a synapomorphy for a genus [18,19], and these regions can sometimes fold into stem–loop secondary structures or hairpin structures. Hairpin structures have been observed at PCG junctions in the mitochondrial genomes of various metazoans, including insects [20,21,22,23,24,25]. Kim et al. [25] suggested that hairpin structures are important for precise cleavage of the mature protein-coding genes. In addition, potential hairpin structures were also found in the A + T-rich region of insect mitogenomes, which was considered to be the initiation site of secondary strand synthesis [21,24,26]. However, the hairpin structures found in insects were relatively short (about 10 bp), and only a few studies have inferred an association between phylogenetic relationships and hairpin structures. We were interested in whether the hairpin structures were associated with synapomorphy at genus or family levels within Microcoryphia. It is well known that the mitogenome is often assumed to be an important neutral marker [27]. However, recent research has shown that the evaluation of selective pressures acting on mitogenome proteins can provide key insights into the adaptive evolution of the mitogenome [28,29,30,31,32,33]. To date, various studies of mammalian [34,35], avian [36], frog [33], fish [37], and insect mitogenomes have indicated that adaptive evolution has occurred. Among insects, this has included studies of Hymenoptera [38], Orthoptera [39], Ephemeroptera [40], Diptera [41], and Lepidoptera [42]. Thus, the mitogenome can be used as a molecular tool to explore/assess adaptive evolution.

In the present study, we sequenced the mitogenomes of *C. coreanus* and *C. songi,* and compared these mitogenomes with those of other bristletails available in the GenBank database. The 13 protein-coding genes were used to construct phylogenetic relationships of Microcoryphia, in order to discuss the relationship of *Coreamachilis*. A positive selection analysis was also used to assess whether *C. coreanus,* with parthenogenic reproduction, and *C. songi,* with sexual reproduction, were under positive selection at the mitogenome level.

## 2. Material and Methods

### 2.1. Specimen Collection and DNA Extraction

The specimens of *C. songi* were collected from Lianyungang city, Jiangsu Province, China, and the specimens of *C. coreanus* were collected from Gongchangling, Liaoyang city, Liaoning Province, China. All specimens were identified by JY Zhang and stored in 100% ethanol in a −40 °C freezer. Total DNA was extracted from individual female specimens using QIAGEN DNeasy blood and tissue kit (QIAGEN, Hilden, Germany).

### 2.2. Polymerase Chain Reaction (PCR) Amplification and Sequencing

After modifying the primers designed by Simon et al. [43] and Ma et al. [44] in combination with published sequences of Machilinae, we designed 15 pairs of universal primers for amplification of mitogenomes (Table 1). PCR was performed using a BioRAD MJ mini personal thermal cycler (California, USA). Based on the sequence information obtained from earlier PCR runs using universal primers, several pairs of specific primers were then designed to obtain the whole mitogenome. Both PCR amplifications and reaction volume were carried out using methods described previously [17]. All PCR products were sequenced in both directions by Sangon Biotech Company (Shanghai, China).

### 2.3. Sequence Analyses and Annotation

Raw sequence files were proofread and assembled using SeqMan in DNASTAR package v.6.0 [45]. The tRNA genes were identified and their cloverleaf secondary structures were determined by MITOS [46]. The locations of the 13 protein-coding genes (PCGs) and two rRNA genes were determined by comparing with homologous sequences of other Microcoryphia mitogenomes [44,47], and the 13 PCGs were translated into amino acids by MEGA 7.0 [48]. The codon skews, relative synonymous codon usage (RSCU), nucleotide composition, and AT and GC skews were calculated using Phylosuite v1.1.16 [49]. Two mitogenome maps were drawn using CGView server V 1.0 [50]. Hairpin structures were identified using RNA secondary structure prediction (http://www.genebee.msu.su/services/rna2_reduced.html, accessed on 26 April 2021) [51].

### 2.4. Phylogenetic Analyses of Microcoryphia

Since Microcoryphia is located at the base of the class Insecta, we chose *Xibalbanus tulumensis* (Nectiopoda: Speleonectidae) [52] and *Daphnia magna* (Anomopoda: Daphniidae) (MT199637) as the outgroups for phylogenetic analyses in this study. To date, only 10 mitogenomes of Microcoryphia were available for download from the NCBI [44,47,53,54,55,56]. Therefore, we combined all mitogenomes of Microcoryphia as the ingroup including *C. coreanus* and *C. songi* along with the two outgroups in order to discuss the phylogenetic relationships in Microcoryphia. Accession numbers of all bristletail mitogenomes are listed in Table 2. Sequences of the thirteen PCGs were extracted from the mitogenomes using PhyloSuite v1.1.16 [49], and aligned in batches with MAFFT integrated into PhyloSuite v1.1.16 using codon-alignment mode. Gblocks in PhyloSuite v1.1.16 was used to remove ambiguous sites. Finally, the 13 protein-coding genes were linked into a single line using concatenate sequence in PhyloSuite v1.1.16. We used the program PartionFinder 1.1.1 [57] to estimate the best partitioning scheme and model according to the Bayesian information criterion (BIC). ML analysis was conducted in RAxML 8.2.0 [58] with the best model of GTRGAMMA and 1000 bootstrap replications. BI analysis was conducted using the MrBayes 3.1 [59] with the model of GTR + I + G set for 10 million generations with sampling every 1000 generations. The first 25% of generations were discarded as burn-in and the average standard deviation of split frequencies in MrBayes 3.1 was below 0.01.

### 2.5. Selection Pressure Analyses of Coreamachilis

The ratio of non-synonymous-to-synonymous (*d_n_*/*d_s_*) ω can indicate selection pressure at the protein level, with ω = 1, ω > 1 and ω < 1 meaning neutral selection, positive selection and negative selection, respectively [60]. The ω value was calculated by the codon-based maximum likelihood method using the CodeML algorithm [60] implemented in the EasyCodeML [61]. Four different models were used to test the selection pressure, including the site model, clade model, branch model and branch-site model. We chose *C. coreanus* and *C. songi* as the foreground branch, respectively, and others as the background branch using three different model selections (the clade model, branch model and branch-site model). The branch models were performed under the one-ratio model (M0) or the two-radio model, the former presuming that ω in specific branches was different from the rest of the tree. Also, the branch-site models were combined with heterogeneous ω across sites and branches (model A) and can be compared against a null model (model A null), which made it possible to find neutral evolution and negative selection. Likelihood ratio tests (LRTs) and Bayes empirical Bayes (BEB) were used to assess these models and evaluate the posterior probability of positive selection sites, respectively. The likelihood ratio test (LRT) was used to compare the statistical model in order to determine whether there were differences between them.

## 3. Results and Discussion

### 3.1. Specimen Collection and Mitogenome Structure

We gathered over three hundred *C. coreanus* female individuals in our collection area, Liaoyang city, Liaoning Province, China, between 2005 and 2015, finding no males among these specimens. Similarly, Mendes [3] collected *C. coreanus* species in North Korea, but did not find any male individuals. Hence, it can be inferred that parthenogenic reproduction has emerged in *C. coreanus*. Only two species of Meinertellidae [62], two species of Petrobiinae [63], and several species of Machilinae [1,64] were previously found to exhibit parthenogenesis. In addition, we collected male and female individuals of *C. Songi*, indicating that it was indeed using the sexual reproduction strategy as reported [4,5].

**Figure 1:**
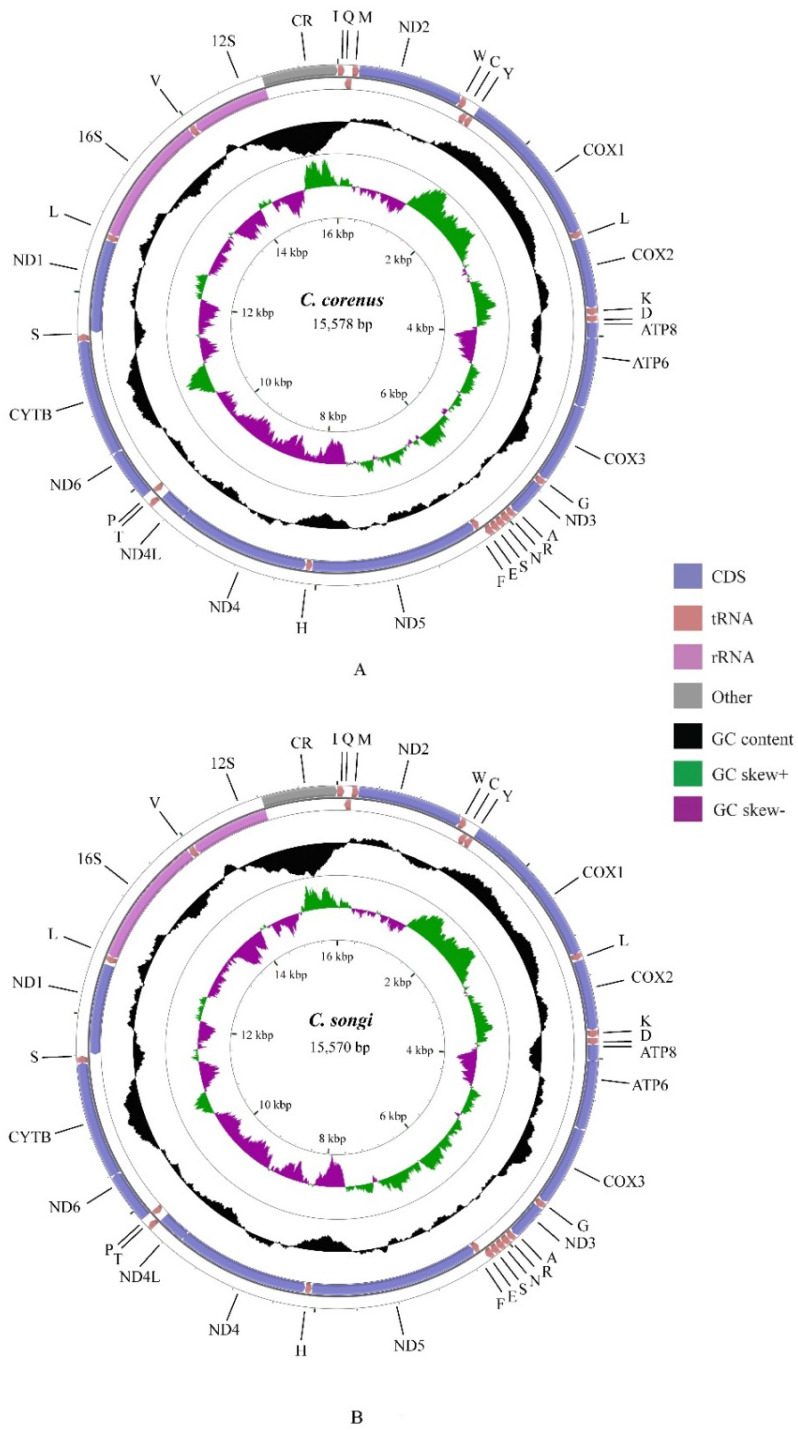
Mitochondrial genome maps of *C. coreanus* (**A**) and *C. songi* (**B**). The first circle shows the gene map (PCGs, rRNAs, tRNAs and the AT-rich region) and the genes outside the map are coded on the majority strand (J-strand) whereas the genes inside the map are coded on the minority strand (N-strand). The second circle shows the GC content and the third shows the GC skew. GC content and GC skew are plotted as the deviation from the average value of the entire sequence.

The complete mitogenomes of *C. coreanus* and *C. songi* were circular DNA molecules of 15,578 bp and 15,570 bp in length, respectively (Table 3). By comparison, the other 10 sequenced mitogenomes of Microcoryphia ranged from 15,473 bp [47] to 16,197 bp [56]. Both the *C. coreanus* and *C. songi* genomes encoded 13 PCGs, two rRNA genes, and 22 tRNA genes (Figure 1), which is consistent with typical insect mitogenomes [65]. Among these, 23 genes were located on the heavy (H) strand and the other 14 genes were on the light (L) strand (Table 3). The gene order of the *C. coreanus* and *C. songi* mitogenomes were the same as those of typical insects. Among the 12 mitogenomes of Microcoryphia, including the two mitogenomes in this study, ten of them had the same gene arrangement, except for *Petrobius brevistylis* [55] and *Trigoniophthalmus alternatus* [56]. In the mitogenomes of *C. coreanus* and *C. songi*, the tRNA^Ala^–tRNA^Arg^–tRNA^Asn^–tRNA^Ser1^–tRNA^Glu^–tRNA^Phe^ cluster was found between ND3 and ND5 genes (Figure 1), as also occurred in eight other mitogenomes of bristletails, except for *P. brevistylis* [55], which showed a cluster of tRNA^Arg^–tRNA^Asn^–tRNA^Ser1^–tRNA^Glu^–tRNA^Ala^–tRNA^Phe^ and *T. alternatus* with a tRNA^Ala^–tRNA^Arg^–tRNA^Asn^–tRNA^Ser1^–tRNA^Glu^–tRNA^Tyr^–tRNA^Phe^ cluster.

There were some overlaps (34 bp and 35 bp) and intergenic regions (37 bp and 60 bp) in the mitogenomes of *C. coreanus* and *C. songi*, respectively. Among other Microcoryphia mitogenomes, the overlaps ranged from 13 bp to 38 bp, and the intergenic regions ranged from 77 bp to 230 bp. The intergenic region of *C. coreanus* was the shortest among the sequenced Microcoryphia, largely due to the short intergenic region between ND1 and tRNA^Ser2^, which was always longer than 20 bp in most of the Microcoryphia species, but was only 6 bp in *C. coreanus*.

The nucleotide composition of the *C. songi* mitogenome was A = 36.38%, T = 35.33%, C = 16.68%, and G = 11.61%, and was very similar to *C. coreanus*, which was as A = 36.67%, T = 35.37%, C = 16.66%, and G = 11.30%. There were strong A + T biases in both *C. songi* and *C. coreanus*, 71.7% and 72.1%, respectively, and these were within the range of other Microcoryphia mitogenomes (67.9–74.4%). The skew statistics showed that there was a positive AT skew and negative GC skew in both *C. coreanus* and *C. songi* (Table 4).

### 3.2. Protein-Codon Genes and Codon Usages

There were 13 PCGs identified in the mitogenomes of *C. coreanus* and *C. songi*, and nine of them were located on the heavy strand (H-strand), whereas the others were on the light strand (L-strand) (Figure 1). Most of the PCGs used ATN (N represents A, G, C, or T) as the start codon, except for ND2 in *C. coreanus*, which used TTG as the start codon (Table 3). Although ATN has been accepted as the canonical mitochondrial start codon for insect mitogenomes [65], TTG is also regarded as a start codon [66]. In fact, before being found in *C. coreanus*, TTG had only been found for the COX1 gene of the *T. alternatus* mitogenome in the Microcoryphia order [56]. In the *C. coreanus* mitogenome, there were seven genes that used ATG as the start codon, which was the same as in *C. songi*. Four genes used ATT as the start codon in *C. coreanus* and five genes used ATT in *C. songi*. The COX3 gene of *C. coreanus* and *C. songi* used ATA as the start codon (Table 5).

The stop codon usage was the same in both *C. coreanus* and *C. songi*. Eleven genes used ATT as the stop codon, whereas an incomplete stop codon (T) was used in COX1 and ND1 genes (Table 5). It is common, in metazoan mitochondrial genomes, to see an incomplete stop codon, and these truncated stop codons are presumed to be completed by post-transcriptional polyadenylation [67].

**Figure 2:**
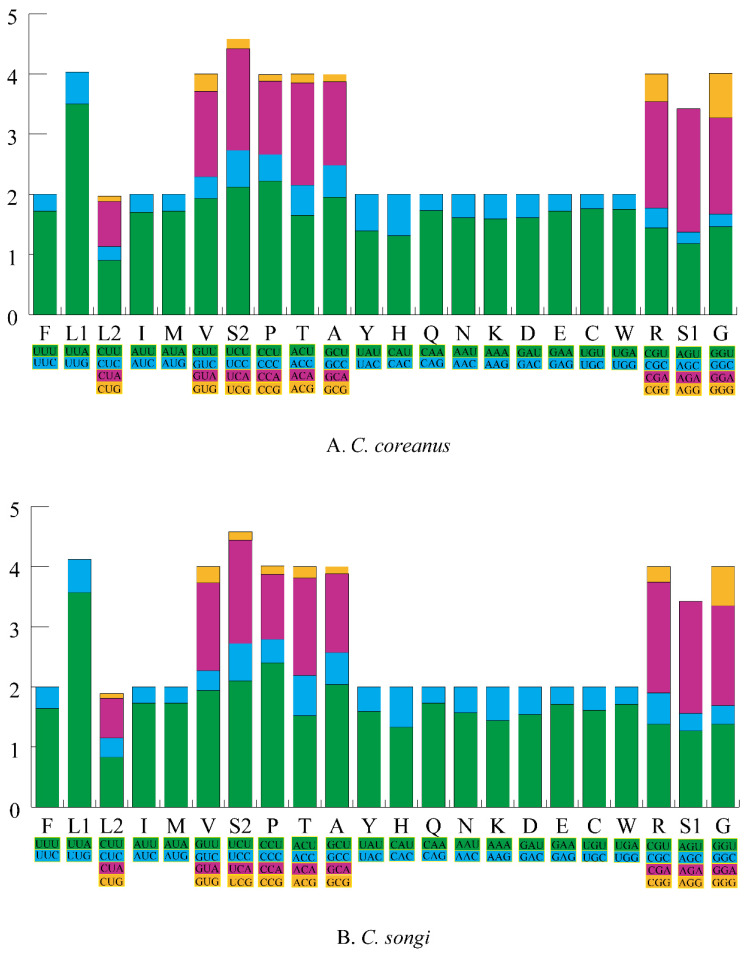
The relative synonymous codon usage (RSCU) in two Microcoryphia mitogenomes. Codon families are provided on the x-axis and the different combinations of synonymous codons that code for each amino acid are defined on the y-axis.

The A + T content of the 13 PCGs was 69.1% and 69% in *C. coreanus* and *C. songi*, respectively. The AT skew and GC skew were negative in both the species (Table 4). The RSCU of *C. coreanus* and *C. songi* is shown in Figure 2. The most frequently encoded amino acids were Leu (UUR), Phe, and Ile (>300), whereas the least frequently used amino acid was Cys (<45).

### 3.3. Ribosomal RNAs, Transfer RNAs and Hairpin Structures

Of the 22 tRNA genes, 14 tRNAs were encoded on the heavy strand (H-strand), whereas the other eight tRNAs were encoded on the light strand (L-strand). Both rRNAs were on the light strand (Table 3). As in other Microcoryphia mitogenomes, the 16S rRNA gene was located between tRNA^Leu^ and tRNA^Val^, with a length of 1332 bp and 1322 bp in *C. coreanus* and *C. songi*, respectively. Located between tRNA^Val^ and the CR, the 12S rRNA gene was 813 bp and 806 bp in *C. coreanus* and *C. songi*, respectively.

The total length of the tRNAs was 938 bp and 1001 bp, with an average A + T content of 74% and 73.4% in *C. coreanus* and *C. songi*, respectively. In both the mitogenomes, most of the tRNA genes displayed the common cloverleaf secondary structure, except for tRNA^Ser^ (AGN), which had lost the dihydrouridine (DHU) arm (Figure S1), as often found in other insect mitogenomes [44].

**Figure 3:**
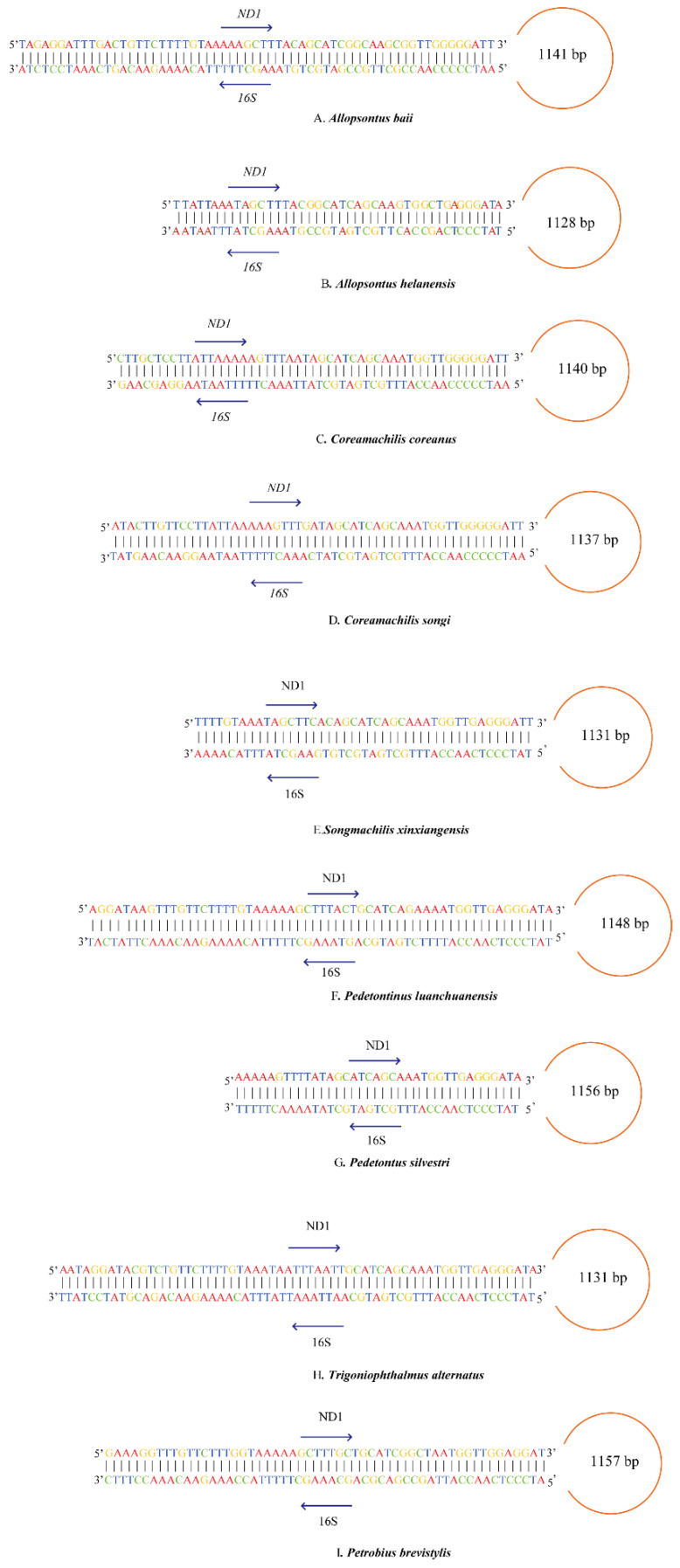
Inferred hairpin structures of nine mitogenomes. The circle means the length between the ND1 gene and 16S RNA gene.

**Figure 4:**
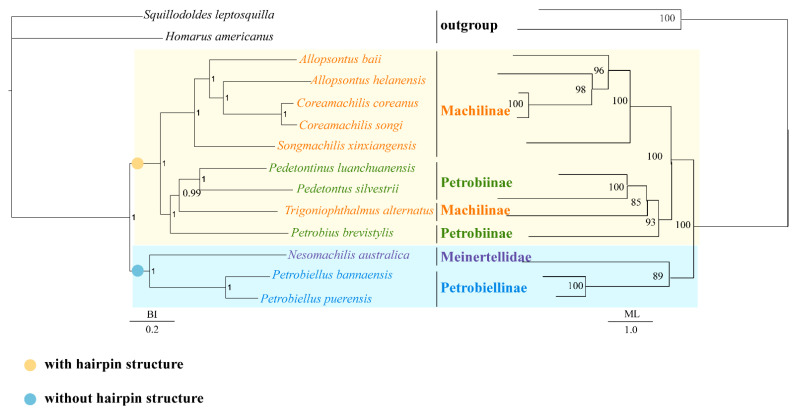
Phylogenetic relationships of *Coreamachilis* inferred from BI analysis (**left**) and ML analysis (**right**) based on 13 mitochondrial protein-coding genes including 12 Microcoryphia species. *H. americanus* and *S. leptosquilla* were used as the outgroup. The numbers above branches specify posterior probabilities as determined from BI (**left**) and bootstrap percentages from ML (**right**).

A novel hairpin structure was located in the ND1 and 16S rRNA genes, with a length of about 60 bp in the mitogenomes of both (Figure 3C,D). In fact, we found a similar hairpin structure in 7 out of the 10 known bristletails mitogenomes (Figure 3A,B,E–I). Some hairpin structures have been found in other species, but all of them were short (less than 10 bp) and mostly located between PCGs [20,21,22,23,24,25]. Such sequences were suggested to have a potential role as a secondary strand-replication origin [25]. In this case, the stop codon of one of the PCGs was incomplete (T or TA), and the 3′-end region of this gene has the potential to form a hairpin structure with the beginning region of the neighboring protein-coding gene, which is important for the precise cleavage of the mature protein-coding genes [67,68,69]. In the mitogenomes of *C. coreanus* and *C. songi,* the stop codon of ND1 was incomplete, but the stem of the hairpin structure in the 16S gene was not at the beginning. Also, there were similar hairpin structures in other bristletail mitogenomes, but all the stop codons of ND1 in those mitogenomes were complete (Table 4), and the stems of the hairpin structures were not at the 3′-end region or beginning region. As for other genes with incomplete stop codons, we did not find any other long hairpin structures like this in other insect orders. In the A + T-rich region, it was reported that stem–loop structures could be formed. Also, it has been mentioned that conserved regions of single stem–loop structure (the 5′ flanking sequences with “TTATA”, while 3′ flanking sequences with a “G(A)nT” motif) have been observed in a variety of insect orders [24,26]. This stem–loop structure in the A + T-rich region was suggested as the site of the initiation of secondary strand synthesis [21,24,26]. However, the hairpin structures we found were not in the control region, and none of the conserved structure was found. This hairpin structure between the ND1 and 16S RNA genes, first reported in Microcoryphia, appears to be novel and about 60 bp in length, with certain homologous segments (Figure 4), unlike the short hairpin structures previously found. No relevant studies about such a structure were found during an online search of the literature, and if a similar hairpin structure can be found in other species, it would be interesting to explore its function.

### 3.4. A + T Rich Region

The largest non-coding region of bristletail mitogenomes was the control region, which was located between the *12S rRNA* and tRNA^Ile^ genes. The length of the A + T-rich region of *C. coreanus* and *C. songi* mtDNA was 736 bp and 734 bp, respectively. Ten other Microcoryphia mitogenomes, published online, showed lengths of the A + T-rich region ranging from 538 bp (*N. australica*) to 1149 bp (*T. alternatus*).

### 3.5. Phylogenetic Analyses and Selection Pressure Analyses

**Figure 5:**
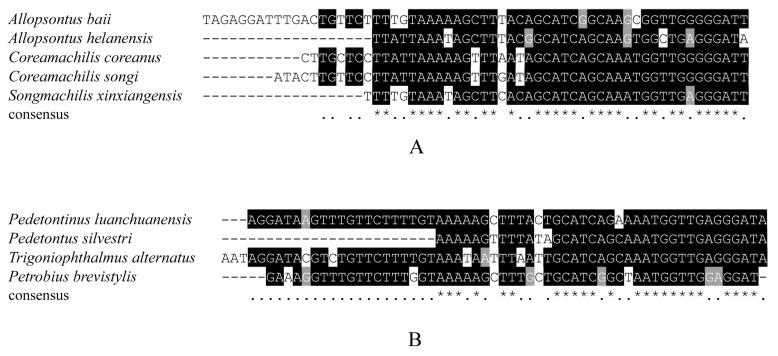
(**A**) Alignment of the stem among *A. baii, A. helanensis, C. coreanus, C. songi* and *S. xinxiangensis*. (**B**) Alignment of the stem among *P. luanchuanensis, P. silvestri, T. alternatus* and *P. brevistylis*.

We constructed BI and ML trees with the nucleotide sequences of the 13 mitochondrial protein-coding genes, including 12 Microcoryphia species and two outgroup species (*X. tulumensis* and *D. magna),* to perform phylogenetic analyses (Figure 4). The topologies of BI and ML analyses were the same. The monophyly of Machilidae failed to be supported because Meinertellidae was clustered into Machilidae and formed a sister clade to Petrobiellinae. The monophyly of Machilinae and Petrobiinae also failed to be supported, because *T. alternatus,* belonging to Machilinae, was clustered into the subfamily Petrobiinae (Figure 4). Similar results were also presented in the study by Ma et al. [44]. The previous main diagnosis of the Machilinae subfamily was via morphological characters of plesiomorphies [1], but our phylogenetic tree-based result was not congruent with this morphological taxonomy. The classification of *Trigoniophthalmus* genus should be reconsidered in combination with phylogenetic trees and morphological characteristics. The clade consisting of (*Songmachilis xinxiangensis* + (*Allopsontus baii* + (*A. helanensis* + (*C. coreanus* + *C. songi*)))) strongly supported the non-monophyly of *Allopsontus* and the monophyly of *Coreamachilis* in BI and ML analyses. The genus *Coreamachilis* was proposed as a monophyletic group, which is consistent with the morphological taxonomy, as well as sequence analysis of COX1 from the mitogenome [70]. However, the paraphyly of *Allopsontus* suggested that the subgenera of *Allopsontus* should be further assessed, because *A. baii* and *A. helanensis* belong to the genus *Allopsontus* s. s. Silvestri, 1911 and *Allopsonsus* (*Anisopsontus*) Mendes, 1990 [71]. Our results suggest that the morphological classification at the genus level can be further improved by combining phylogenetic relationships. It was interesting that the hairpin structure between ND1 and 16S RNA genes is a synapomorphy of the clade including Machilinae and Petrobiinae. (Figure 3). In addition, in the clade with the long hairpin structure, the homologous segments of hairpin structures aggregated together are more alike (Figure 4). The five species of Machilinae were grouped into a branch, and their stem sequences in the hairpin structure showed a high similarity (Figure 5A). At the same time, the remaining clades with a hairpin structure were also of relative similarity (Figure 5B). It is well known that gene order and genome organization can provide useful genetic information to understand evolutionary relationships [72]. Hence, intergenic regions can be used as a synapomorphy for a genus [18,19]. The hairpin structure found in this study is located in the gene spacer between ND1 and 16S, and as a special structure, it is highly correlated with the results of the phylogenetic tree. Hence, we indicate that the long hairpin structure may be useful as a molecular characteristic to discuss phylogenetic relationships within Microcoryphia.

The phylogenetic tree developed from the current data was used for selection pressure analyses. Four different models were used in the test, including the site model (M3 vs. M0, M1a vs. M2a, M7 vs. M8, and M8 vs. M8a), clade model (CmC vs. M2a_rel), branch model (two-ratio model vs. M0), and branch-site model (A vs. A null) (Table S1). We chose *Coreamachilis* as the foreground branch and other species as the background branch in the clade model, branch model, and branch-site model. However, the LRTs did not indicate any sign of positive selection (*p* > 0.05). Since *C. coreanus* and *C. songi* reproduced differently, we calculated the selective pressures on each of them, to see if there was an adaptive evolution at the mitochondrial gene level for the differences in reproductive patterns. However, the results did not indicate any sign of positive selection either (Tables S2 and S3). Neither *C. coreanus* (with parthenogenesis) nor *C. songi* (with sexual reproduction) were subject to any positive selection. In a word, it was suggested that differences in *Coreamachilis* reproductive patterns may not result from adaptive evolution on the mitogenome.

## 4. Conclusions

We successfully sequenced the complete mitogenomes of *C. coreanus* and *C. songi*, which showed similar gene characteristics to the other 10 species of Microcoryphia with published mitogenomes. We found a special long hairpin structure (about 60 bp) located between ND1 and 16S RNA genes of *C. coreanus* and *C. songi*, which was also found in seven known bristletail mitogenomes belonging to Petrobiinae and Machilinae. However, the function and formation of this long hairpin structure remain unclear, and still need to be explored. Since the hairpin structure is highly correlated with the phylogenetic tree, the long hairpin structure can be used as the synapomorphy for Machilinae and Petrobiinae. Both BI and ML analyses supported the genus *Coreamachilis* as a monophyletic group, whereas the monophyly of *Allopsontus* was not recovered. Neither *C. coreanus* (with parthenogenesis) nor *C. songi* (with sexual reproduction) were subject to any positive selection.

## Figures and Tables

**Table 1:** List of universal primers used for PCR amplification according to Simon et al. [43].

Number	Primer Name	Sequence (5′-3′)	Length	Target Gene Region
1	SB-J-165	AAGCTANTGGGYTCATAYCC	1000 bp	tRNA^Met^-ND2
	SB-N-1273	CAGCTTTGAAGGCTAWTAGTTT		
2	SB-J-20	GGATTACARTGATAAAGTAAA	3000 bp	tRNA^Ile^-COX1
	SB-N-3003	TCCRATGCACTTWTCTGCCAAAWTA		
3	SB-J-2195	TGATTYTTTGGNCAYCCHGAAGT	1000 bp	COX1
	SB-N-3014	TCCRATGCACTTATCTGCCAARTTA		
4	SB-J-2161	TATTTTGATTYTTTGGNCAYCCHGAAGT	1500 bp	COX1-COX2
	SB-N-3646	CCACARATTTCNGARCAYTG		
5	SB-J-3360	ACWATHGGDCAYCAATGATAYTG	1300 bp	COX2-ATP8
	SB-N-4061	GARAATARGTTDGTTATCATTTTCA		
6	SB-J-3645	GGCCAATGYTCNGAAATYTGYGG	3800 bp	COX2-ND5
	SB-N-7462	CCWGCDGCTATRGCHGCNCC		
7	SB-J-5747	CCATTYGAATGYGGNTTTGAYCC	500 bp	ND3-tRNA^Asn^
	SB-N-6160	CTTAATRDTABCATTAACAGTGR		
8	J-12517	CGGTTTCAACTCAGATCATGTA	800 bp	ND1-16S rRNA
	N-13321	CACCTGCTTATCAAAAACA		
9	SB-J-7077	ATYAAATCYTTWGARTAAAAHCC	700 bp	ND5
	SB-N-7793	TTDGGDTGRGATGGDTTDGG		
10	SB-J-7572	AAADGGAATTTGDGCTGTYTTAGT	1200 bp	ND5-ND4
	SB-N-8727	AARGCDTTAATTGCBTAYTCWTC		
11	SB-J-8641	CCWCTHGARCAYAANCCATG	500 bp	ND4
	SB-N-9153	TGRGGRTATCARCCWGARCG		
12	SB-J-8882	GGHGCTTCNACRTGAGCYTT	2000 bp	ND4-Cyt *b*
	SB-N-10885	CCTCARAANGATATYTGHCCTCA		
13	SB-J-10690	TGYCGAGATGTWAATTAYGGWTG	1800 bp	Cyt *b*-ND1
	SB-N-12489	TATRTTCARATTCGDAAAGGDCC		
14	SB-J-12887	CCGGTYTGAACTCAGATCATGT	500 bp	16S rRNA
	SB-N-13398	CGCCTGTTTAYCAAAAACATGKC		
15	SB-J-10873	TATGTTYTHCCNTGAGGDCAAATRTC	3700 bp	Cyt *b*-CR
	SB-N-14556	TAAACTAGGATTAGATACCCTATTAT		

**Table 2:** Species used to construct the phylogenetic relationships along with GenBank accession numbers.

Order	Family	Species	GenBank Accession Number	References
Nectiopoda	Speleonectidae	*Xibalbanus tulumensis*	NC_005938	[52]
Anomopoda	Daphniidae	*Daphnia magna*	MT199637	Unpublished
Microcoryphia	Meinertellidae	*Nesomachilis australica*	AY793551	[53]
	Machilidae	*Allopsontus baii*	KJ754500	[44]
		*Allopsontus helanensis*	KJ754501	[44]
		*Coreamachilis songi*	MW752138	This study
		*Coreamachilis coreanus*	MW752137	This study
		*Pedetontus silvestrii*	EU621793	[54]
		*Pedetontinus luanchuanensis*	KJ754502	[44]
		*Petrobiellus bannaensis*	KJ754503	[44]
		*Petrobiellus puerensis*	KJ754504	[44]
		*Petrobius brevistylis*	AY956355	[55]
		*Songmachilis xinxiangensis*	JX308221	[47]
		*Trigoniophthalmus alternatus*	EU016193	[56]

**Table 3:** Gene arrangement of *C. coreanus* (*C. c*) and *C. songi* (*C. s*) mitochondrial genome.

Gene	Strand	Position		Start Codon	Stop Codon
		*C. c*	*C. s*	*C. c*/*C. s*	*C. c*/*C. s*
tRNA^Ile^	H	1–67	1–67		
tRNA^Gln^	L	68–138	68–137		
tRNA^Met^	H	145–210	143–208		
ND2	H	211–1239	208–1236	TTG/ATG	TAA/TAA
tRNA^Trp^	H	1239–1304	1236–1301		
tRNA^Cys^	L	1304–1370	1301–1368		
tRNA^Tyr^	L	1371–1438	1369–1436		
COX1	H	1431–2975	1429–2973	ATT/ATT	TAA/TAA
tRNA^Leu2(UUA)^	H	2971–3035	2969–3033		
COX2	H	3042–3729	3040–3727	ATG/ATG	T/T
tRNA^Lys^	H	3730–3800	3728–3798		
tRNA^Asp^	H	3804–3871	3803–3870		
ATP8	H	3872–4033	3871–4032	ATT/ATT	TAA/TAA
ATP6	H	4027–4704	4026–4703	ATT/ATT	TAA/TAA
COX3	H	4704–5486	4703–5485	ATG/ATG	TAA/TAA
tRNA^Gly^	H	5489–5556	5488–5554		
ND3	H	5557–5910	5555–5908	ATC/ATC	TAA/TAA
tRNA^Ala^	H	5914–5976	5912–5974		
tRNA^Arg^	H	5979–6049	5977–6045		
tRNA^Asn^	H	6050–6116	6046–6112		
tRNA^Ser1^	H	6117–6183	6113–6179		
tRNA^Glu^	H	6184–6252	6180–6248		
tRNA^Phe^	L	6254–6318	6250–6314		
ND5	L	6318–8048	6315–8045	ATG/ATG	TAA/TAA
tRNA^His^	L	8050–8113	8047–8110		
ND4	L	8118–9467	8115–9464	ATG/ATG	TAA/TAA
ND4L	L	9461–9760	9458–9757	ATG/ATG	TAA/TAA
tRNA^Thr^	H	9763–9824	9760–9821		
tRNA^Pro^	L	9825–9887	9822–9885		
ND6	H	9891–10,397	9889–10,395	ATT/ATT	TAA/TAA
Cyt *b*	H	10,397–11,533	10,395–11,531	ATG/ATG	TAA/TAA
tRNA^Ser2^	H	11,532–11,599	11,530–11,597		
ND1	L	11,606–12,554	11,604–12,531	ATG/ATT	T/T
tRNA^Leu1(CUA)^	L	12,555–12,620	12,553–12,618		
16S rRNA	L	12,621–13,959	12,619–13,953		
tRNA^Val^	L	13,960–14,032	13,954–14,026		
12S rRNA	L	14,033–14,841	14,027–14,836		
CR		14,842–15,578	14,837–15,570		

**Table 4:** Base composition, the percent of A and T, AT skew and GC skew of *C. coreanus* and *C. songi*.

Region	*C. coreanus*				*C. songi*			
	Length (bp)	AT%	AT Skew	GC Skew	Length (bp)	AT%	AT Skew	GC Skew
Whole genome	15,578	72.1	−0.018	−0.191	15,570	71.7	−0.015	−0.179
Protein-coding genes	6882	69.1	−0.121	−0.158	6882	69	−0.125	−0.156
Ribosomal RNA genes	2148	74	−0.021	−0.282	2145	73.7	−0.023	−0.285
Transfer RNA	938	74	−0.029	−0.016	1001	73.4	−0.011	−0.011

**Table 5:** Start and stop codons of the Microcoryphia mitochondrial genomes. Notes: *Allopsonsu baii* (*A. b*); *Allopsonsus helanensis (A. he)*; *Coreamachilis coreanus (C. c)*; *Coreamachilis songi (C. s)*; *Nesomachilis australica (N. a)*; *Petrobius brevistylis (P. b)*; *Petrobiellus bannaensis (P. ba)*; *Pedetontinus luanchuanensis (P. l)*; *Petrobiellus puerensis (P. p)*; *Pedetontus silvestrii (P. si)*; *Songmachilis xinxiangensis (S. x)*; *Trigoniophthalmus alternatus (T. a)*.

Gene	Start Codon/Stop Codon											
	*A. b*	*A. he*	*C. c*	*C. s*	*N. a*	*P. b*	*P. ba*	*P. l*	*P. p*	*P. si*	*S. x*	*T. a*
ATP6	ATA/TAA	ATG/TAA	ATT/TAA	ATT/TAA	ATA/TAA	ATG/TAA	ATG/TAA	ATG/TAA	ATG/TAA	ATG/TAA	ATG/TAA	ATG/TAA
ATP8	ATT/TAA	ATT/TAA	ATT/TAA	ATT/TAA	ATA/T	ATT/TAA	ATC/TAA	ATT/TAA	ATC/TAA	ATT/TAA	ATT/TAA	ATT/TAA
COX1	ATT/T	ATT/T	ATT/TAA	ATT/TAA	ATT/TA	ATG/TAA	ATT/TAA	ATT/T	ATT/TAA	ATT/T	TCG/T	TTG/TA
COX2	ATG/T	ATG/T	ATG/T	ATG/T	ATG/T	ATG/T	ATG/TAA	ATG/T	GTG/TAA	ATT/T	ATG/T	ATG/TAA
COX3	ATA/TAA	ATG/TAA	ATG/TAA	ATG/TAA	ATG/TAA	ATG/TAA	ATA/TAA	ATG/TA	ATG/TAA	ATG/T	ATG/TAA	ATG/TAA
Cyt *b*	ATG/TAA	ATG/TAA	ATG/TAA	ATG/TAA	ATG/TAA	ATG/TAA	ATG/TAA	ATG/TAA	ATG/TAA	ATG/TAA	ATG/TAA	ATG/TAA
ND1	ATT/TAG	GTG/TAA	ATG/T	ATT/T	ATA/TAA	ATA/T	ATA/TAG	ATT/TAG	ATT/TAA	ATT/TAG	ATT/TAA	ATG/TAA
ND2	ATT/TAA	ATC/TAA	TTG/TAA	ATG/TAA	ATC/TAA	ATT/TAA	ATA/TAA	ATG/TAA	ATA/TAG	ATT/TAA	ATA/TAA	ATG/TAA
ND3	ATA/TAA	ATA/TAA	ATA/TAA	ATA/TAA	ATA/TAA	ATT/TAA	ATA/TAG	ATA/TAA	ATA/TAG	ATA/TAA	ATA/TAA	ATA/TAA
ND4	ATG/TAA	ATT/TAA	ATG/TAA	ATG/TAA	ATT/TAA	ATG/TAA	ATG/TAA	ATG/TAG	ATG/TAG	ATG/TAG	ATG/TAG	ATG/TA
ND4L	ATG/TAA	ATG/TAA	ATG/TAA	ATG/TAA	ATT/TAA	ATG/TAA	ATG/TAA	ATG/TAA	ATG/TAA	ATG/TAA	ATG/TAA	ATG/TAA
ND5	ATG/TA	ATG/TAA	ATG/TAA	ATG/TAA	ATA/TAG	ATG/TAA	ATT/TAG	GTG/T	ATG/TA	ATG/T	ATG/TAA	ATG/TAA
ND6	ATT/TAA	ATT/TAA	ATT/TAA	ATT/TAA	ATT/TAA	ATA/TAA	ATT/TAA	ATT/TAA	ATT/TAA	ATT/TAA	ATT/TAA	ATA/TAA

## Data Availability

The data supporting the findings of this study are openly available in National Center for Biotechnology Information (https://www.ncbi.nlm.nih.gov), accession numbers were MW752137 (*Coreamachilis coreanus*) and MW752138 (*Coreamachilis songi*).

## Supplementary Materials

The following are available online at https://www.mdpi.com/article/10.3390/insects12090795/s1: Figure S1. Inferred secondary structures of the tRNA genes in *C. coreanus* (A) and *C. songi* (B); Table S1. CodeML analysis of mitochondrial protein-encoding genes (13 genes) (choosing *Coreamachilis* as the foreground branch); Table S2. CodeML analysis of mitochondrial protein-encoding genes (13 genes) (choosing *C. songi* as the foreground branch); Table S3. CodeML analysis of mitochondrial protein-encoding genes (13 genes) (choosing *C. coreanus* as the foreground branch).
