# Supplementary material for: Comparative Mitogenomes of Two Coreamachilis Species (Microcoryphia: Machilidae) along with Phylogenetic Analyses of Microcoryphia
Source: Insects. 2021 Sep 5;12(9):795. doi: 10.3390/insects12090795 (PMC8471023; doi:10.3390/insects12090795)
Supplement: Supplementary file 1 [file insects-12-00795-s001.zip › Table S1 selection pressure.pdf]

Table S1 CodeML analysis of mitochondrial protein-encoding genes (13 genes) (*Coreamachilis* as the foreground branch)

| | Model | np | Ln L | Model compared | LRT P-value | Positive sites |
|---|---|---|---|---|---|---|
| Branch model | Two ratio Model 2 | 25 | -85287.650122 | Model 0 vs. Two ratio Model 2 | 0.408895446 | |
| | Model 0 | 24 | -85287.991127 | | | |
| Branch site model | Model A | 27 | -83671.873488 | Model A vs. Model A null | 0.324897750 | 181 G 0.881, 763 A 0.771, 870 L 0.709, 918 V 0.738, 1000 K 0.969*,1014 K 0.509, 1701 S 0.662, 1819 F 0.578, 2005 L 0.624, 2223 L 0.615, 2278 I 0.570, 2356 L 0.509, 2660 G 0.531, 2662 L 0.610, 2671 E 0.858, 3206 P 0.890, 3305 A 0.671, 3353 V 0.782, 3508 L 0.898, 3592 D 0.508, 3603 N 0.844, 3615 S 0.779, 3618 V 0.562 |
| | Model A null | 26 | -83672.358052 | | | Not Allowed |
| Clade model | CmC | 28 | -81557.305666 | M2a_rel vs CmC | 0.000000000 | |
| | M2a_rel | 27 | -81586.915580 | | | Not Allowed |
| Site model | M3 | 28 | -81387.375872 | M0 vs. M3 | 0.000000000 | |
| | M0 | 24 | -85287.991127 | | | Not Allowed |
| | M2a | 27 | -83679.125055 | M1a vs. M2a | 1.000000000 | |
| | M1a | 25 | -83679.125055 | | | Not Allowed |
| | M8 | 27 | -81490.306203 | M7 vs.M8 | 0.000000000 | 51 S 0.550, 256 L 0.747, 905 D 0.686, 2123 M 0.775, 2236 N 0.904, 2256 T 0.839, 2390 V 0.621, 2458 G 0.543, 2534 N 0.522, 2930 L 0.523, 3391 L 0.804, 3394 V 0.747, 3397 L 0.509, 3597 L 0.661 |
| | M7 | 25 | -81533.937285 | | | Not Allowed |
| | M8a | 26 | -81367.132828 | M8a vs.M8 | 0.000000000 | Not Allowed |
