# Supplementary material for: Comparative Mitogenomes of Two Coreamachilis Species (Microcoryphia: Machilidae) along with Phylogenetic Analyses of Microcoryphia
Source: Insects. 2021 Sep 5;12(9):795. doi: 10.3390/insects12090795 (PMC8471023; doi:10.3390/insects12090795)
Supplement: Supplementary file 1 [file insects-12-00795-s001.zip › Table S2 selection pressure songi.pdf]

Table S2 CodeML analysis of mitochondrial protein-encoding genes (13 genes) (chosing *C. songi* as the foreground branch)

	Model	np	Ln L	Model compared	LRT P-value	Positive sites
Branch model	Two ratio Model 2	25	-85235.524365	Model 0 vs. Two ratio Model 2	0.000000000	
	Model 0	24	-85287.991127			
Branch site model	Model A	27	-85235.524365	Model A vs. Model A null	0.000000000	
	Model A null	26	-83679.125056			Not Allowed
Clade model	CmC	28	-81532.325428	M2a_rel vs CmC	0.000000000	
	M2a_rel	27	-81586.915580			Not Allowed
