# Supplementary figures and images for: Comparative Mitogenomes of Two Coreamachilis Species (Microcoryphia: Machilidae) along with Phylogenetic Analyses of Microcoryphia
Source: Insects. 2021 Sep 5;12(9):795. doi: 10.3390/insects12090795 (PMC8471023; doi:10.3390/insects12090795)

A. *C. coreanus*

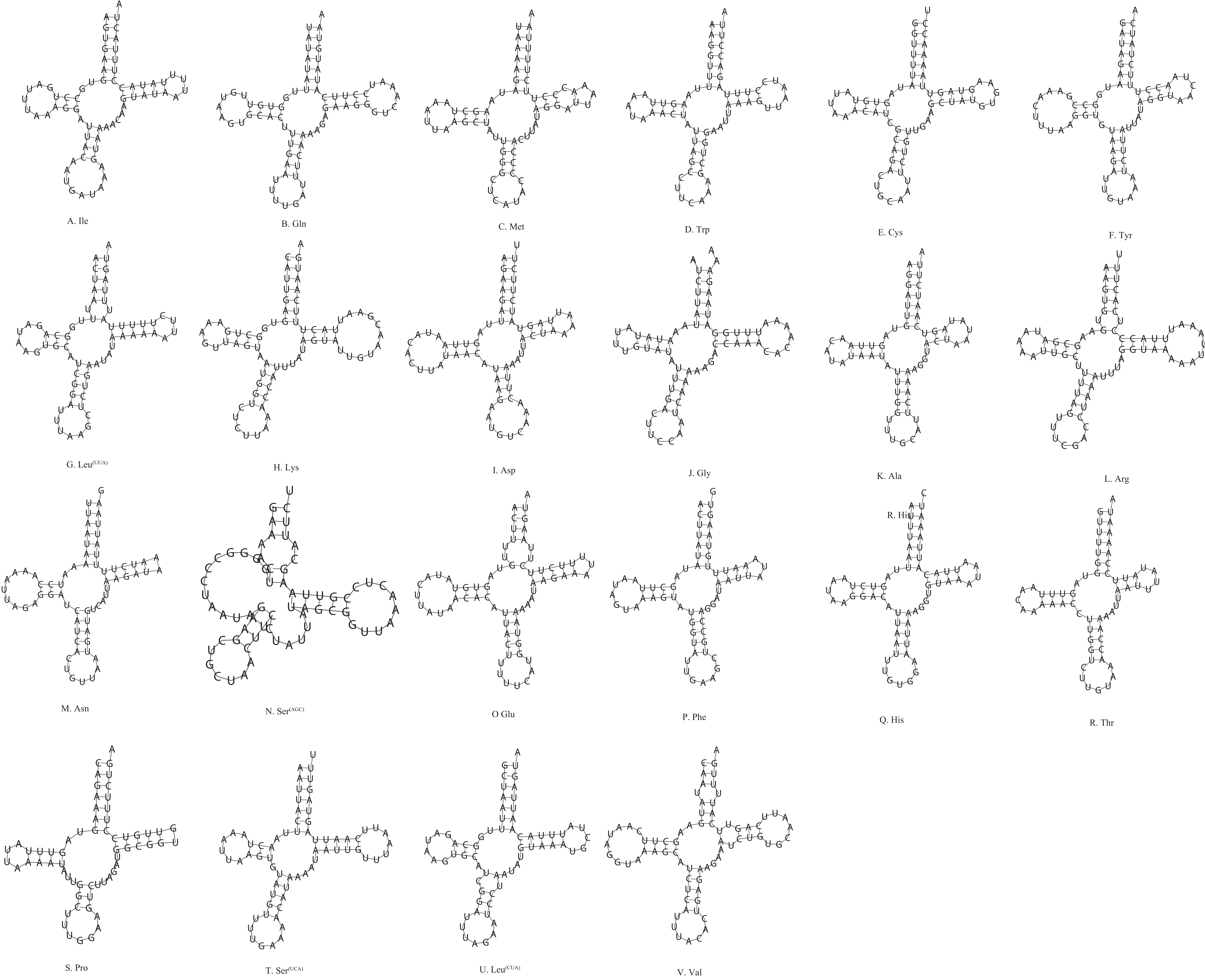

B. *C. songi*

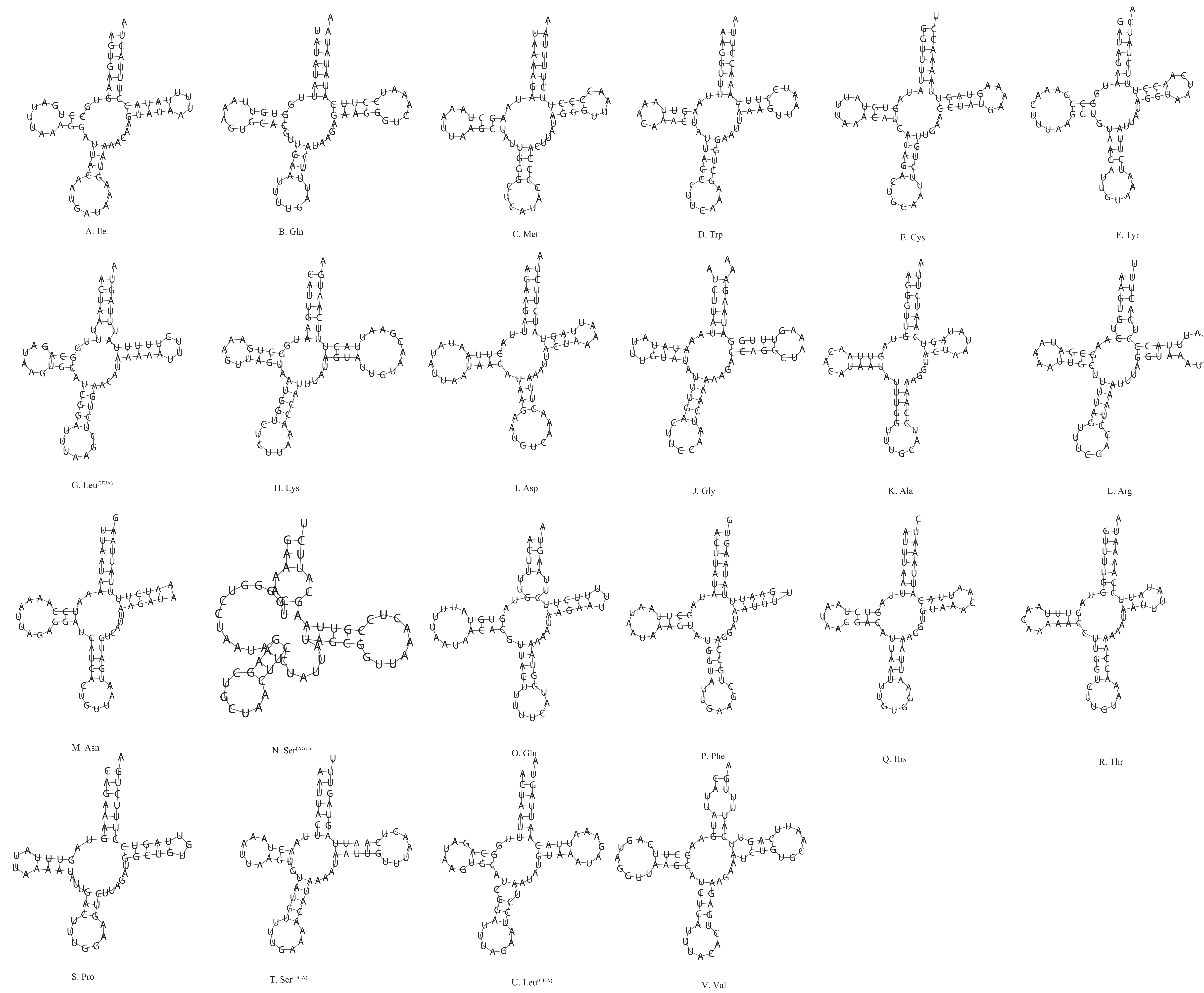

Supplement: Supplementary file 1 [file insects-12-00795-s001.zip › Figure S1 trna.pdf]
